# Joint manifestations revealing inborn metabolic diseases in adults: a narrative review

**DOI:** 10.1186/s13023-023-02810-6

**Published:** 2023-08-10

**Authors:** Amaury Loret, Claire Jacob, Saloua Mammou, Adrien Bigot, Hélène Blasco, Alexandra Audemard-Verger, Ida VD Schwartz, Denis Mulleman, François Maillot

**Affiliations:** 1 Department of Internal Medicine, University Hospital of Tours, Tours, France; 2 Department of Rheumatology, University Hospital of Tours, Tours, France; 3 Biochemistry laboratory, University Hospital of Tours, Tours, France; 4 UMR INSERM 1253, Tours, France; 5 Reference center for inherited metabolic diseases, Tours, France; 6 Medical Genetics Service/Genetics Department, Hospital de Clinicas de Porto Alegre, Universidade Federal do Rio Grande do Sul, Porto Alegre - RS, Brazil; 7 Department of Internal Medicine, Hôpital Bretonneau, 2 Boulevard Tonnellé, CHRU de Tours, Tours cedex, 37044 France

**Keywords:** “Arthralgia”, “Arthritis”, “Joints”, “Inherited metabolic diseases”

## Abstract

Inborn metabolic diseases (IMD) are rare conditions that can be diagnosed during adulthood. Patients with IMD may have joint symptoms and the challenge is to establish an early diagnosis in order to institute appropriate treatment and prevent irreversible damage. This review describes the joint manifestations of IMD that may be encountered in adults. The clinical settings considered were arthralgia and joint stiffness as well as arthritis. Unspecific arthralgias are often the first symptoms of hereditary hemochromatosis, chronic low back pain may reveal an intervertebral disc calcification in relation with alkaptonuria, and progressive joint stiffness may correspond to a mucopolysaccharidosis or mucolipidosis. Gaucher disease is initially revealed by painful acute attacks mimicking joint pain described as “bone crises”. Some IMD may induce microcrystalline arthropathy. Beyond classical gout, there are also gouts in connection with purine metabolism disorders known as “enzymopathic gouts”. Pyrophosphate arthropathy can also be part of the clinical spectrum of Gitelman syndrome or hypophosphatasia. Oxalate crystals arthritis can reveal a primary hyperoxaluria. Destructive arthritis may be indicative of Wilson’s disease. Non-destructive arthritis may be seen in mevalonate kinase deficiency and familial hypercholesterolemia.

**Supplementary Information:**

The online version contains supplementary material available at 10.1186/s13023-023-02810-6.

## Background

Inborn metabolic diseases (IMD) are due, in most cases, to a genetic deficiency of enzymes or transporters, resulting in the disruption of a metabolic pathway leading to synthesis or degradation of organic compounds. These abnormalities may be responsible for the accumulation of toxic substances among blood and organs upstream of the deficiency or downstream of a production defect. IMD are usually inherited in an autosomal recessive mode but the pattern of inheritance may also be X-linked or dominant [1].

IMDs are rare diseases with a large clinical spectrum. They are mostly symptomatic during childhood, mostly in the neonate period or during the first year of life, but some may be diagnosed in adolescence and adulthood [2]. There are also milder forms of IMD which are only detected in adulthood as well as adult-specific forms. Because these diseases are rare and labeled as “pediatric”, physicians are relatively ignorant about their existence in adults and therefore their diagnosis is significantly delayed. In some exceptional cases, some patients are diagnosed with IMD in the elderly [3]. Therapy of many IMD is mainly based on a specific dietary management but recent advances enabled the development of specific therapies such as enzymatic stimulation using co-factors or enzyme replacement therapy and chelating treatments [4].

In adults, most IMD are diagnosed through neuropsychiatric symptoms but some may also be revealed by joint manifestations [5, 6]. Thus, joint manifestations, whether or not associated with other systemic features, can lead the clinician to the diagnosis of IMD. Early diagnosis of these diseases may, when treatable, help in preventing the occurrence of irreversible joint destruction and deformity and influence global outcome for the patients. IMD are unknown from the majority of “non metabolic physicians”, although rheumatologists frequently encounter non-specific clinical manifestations as arthralgia or arthritis that can result in an important diagnosis delay [7].

The present review is part of an approach by physicians to recognize these diseases in adults, by recalling the main IMD with joint damage and proposing a useful diagnostic approach to the clinician. To this end, a literature search in *Medline* including the terms of *Medical Subjects Headings (MeSH)* “arthralgia”, “arthritis”, “joints”, and “metabolism, inborn errors”, with age limit above 19 years, including only French and English texts, was carried out. Given the rarity of some diseases, clinical cases and case studies were kept in our literature review. This review was complemented by reading the main works about IMDs and by experience gained in the IMD reference center of our university hospital over 20 years.

## Main text

### Arthralgia and joint stiffness

Arthralgia or joint stiffness may reveal an IMD in adulthood. They are most often non-specific, with a very broad clinical spectrum of varying severity. Thus, any “unexplained” arthralgia or joint pain occurring at a young age should suggest the diagnosis of IMD. Therefore, etiological orientation requires the family history, age of onset of joint symptoms and their evolution, as well as extra-articular symptoms. A detailed and focused physical examination should be carried out. Some biological and radiological features will help strengthening the etiologic orientation (Table 1).

**Table 1:** IMDs revealed by arthralgia in adulthood: the essential

	Gene	Inheritance	Patients profile rheumatologists should be aware of	Joint manifestations	Treatment
**HFE1 hemochromatosis**	*HFE*	Autosomal recessive with variable penetrance.	Onset after 35 years : Asthenia, increased aminotransferases, arthralgias Melanodermia, diabetes, heart disease, hypogonadism ↑CST, ↑serum ferritin	MCP of the 2nd and 3rd fingers Chondrocalcinosis	Therapeutic bloodletting
**Alkaptonuria-ochronosis**	*HGD*	Autosomal recessive	Onset after 30–40 years : Bluish nose, sclera and ears, black urine, ↑ urinary homogentisic acid	Chronic low back pain, and then arthralgia of the large joints Diffuse calcification of the intervertebral discs	Nitisinone
**MPS**	*IDUA (1)* *IDS (2)* *GALNS (4a)* *ARSB (6)* *GUSB (7)*	Autosomal recessive (except MPS 2, X-linked)	Onset within the 1st decade : Recurrent hernia, corneal clouding, organomegaly, valvular heart disease, sleep apnea syndrome ↑ Urinary GAGs specific enzyme activity	Non-inflammatory progressive stiffness in the hands and shoulders Claw hand, carpal tunnel syndrome, hip dysplasia, multiple dysostosis, spinal deformities	Enzyme replacement therapy (for MPS 1, 2, 4a,6 and 7)
**Type 3 mucolipidosis**	*GNPTAB or GNPTG*	Autosomal recessive	Onset within the 1st decade : organomegaly, valvulopathies Acid hydrolases activity: ↑ in the serum ↓ in cultured fibroblasts	Non-inflammatory progressive stiffness in the hands and shoulders Carpal tunnel syndrome, hip dysplasia, multiple dysostosis, spinal deformities	Symptomatic
**Gaucher disease type 1**	*GBA*	Autosomal recessive	Mean age at diagnosis 22 years : Hepato-splenomegaly, anemia and thrombocytopenia, bone infarction, osteolysis, osteoporosis, small in size ↓ glucocerebrosidase activity	Acute painful crises: aseptic osteonecrosis Chronic pain: arthropathies, deformities	Enzyme replacement therapy or substrate reduction therapy

Fabry disease was not included into this review since pain is neuropathic in this condition and therefore not directly related to joint involvement. Nonetheless, Fabry disease can be a difficult diagnosis that rheumatologists should know [7].

#### Hereditary hemochromatosis

Hereditary Hemochromatosis (HH) are a group of disorders of iron metabolism. The most common form, HFE hemochromatosis (HH type 1), is caused by biallelic variants in the *HFE* gene which encodes the homeostatic iron regulator (HFE), a membrane protein that increases the hepcidin production. The loss of function of HFE causes a reduction in the concentration of hepcidin and, therefore, an increase of intestinal absorption and recycling of iron in macrophages, leading to iron overload/storage in the liver, pancreas, heart, joints and endocrine glands. Transmission is autosomal recessive with variable penetrance. Prevalence of HH is 1/200. The most frequent pathogenic variant is p.Cys282Tyr [8].

Iron overload is determined by the elevation of the coefficient of saturation of transferrin > 45%. The diagnosis of HFE1 hemochromatosis is confirmed by genetic analysis. Iron overload is then assessed by serum ferritin (N < 200 µg/L in women and 300 µg/L in men) and quantitative liver MRI. Treatment is based upon phlebotomy, with a target of serum ferritin < 50 ng/mL. Phlebotomy has little or no effect on articular symptoms but can prevent other organs affection. Thus, treatment of joint manifestations is based on analgesics and non-steroid anti-inflammatory drugs. From the index case, a family investigation must be performed [8, 9].

The first symptoms usually appear after 35 years, after several years of biological iron overload. Chronic fatigue, arthralgia and elevated liver enzymes are the first symptoms of iron overload [10]. In the complete form of HH, clinical symptoms are, in order of frequency : arthralgia, liver disease, skin pigmentation, osteoporosis, diabetes, cardiomyopathy, sexual dysfunction and hypogonadotropic hypogonadism [11].

Arthralgia may be mono, oligo or polyarticular, of varying severity, mechanical or inflammatory, bilateral, affecting any joints. A suggestive presentation can be joint damage affecting a joint usually non affected by primary osteoarthritis, without history of trauma. In order of frequency, the metacarpophalangeal joints, proximal interphalangeal joints, knees, back and neck, feet, hips, wrists, shoulders, distal interphalangeal joints and ankles are involved [12]. However, the most suggestive and almost pathognomonic presentation is the involvement of metacarpophalangeal joints of the second and third fingers, which is characterized initially by a simple painful limitation of flexion referred to as the sign of “the painful handshake” or “iron salute” [13]. A chronic swelling of the 2nd and 3rd metacarpophalangeal joints or a non-inflammatory irreversible deformation may then develop. Such type of arthritis is found to occur in 37.6% of the patients with HH and is associated with age, male gender, hyperferritinemia, diabetes and hepatic cirrhosis [14]. The other joint manifestation linked to HH is the occurrence of acute attacks of pyrophosphate arthropathy, formerly chondrocalcinosis, concerning 1 patient out of 3 [15]. Damage to the hips and knees is the most disabling, with a 2 to 3-fold increased prevalence of prosthetic replacements as compared to the general population [16].

X-rays show geodes encircled by condensation and positioned in the subchondral bone area, osteophytes with rounded edges or hook-like osteophytes, joint space narrowing as well as chondrocalcinosis of wrists, hips and knees joints [17].

The non-specific presentation often results in late diagnosis, although early diagnosis would help prevent organ damages due to the disease. Thus, the presence of arthralgia in a young person should readily lead the clinician to suspect HH and, in these cases, carry out an iron assessment.

#### Alkaptonuria-ochronosis

Alkaptonuria (AKU) was the first IMD to be described by Garrod in 1902. It is caused by biallelic pathogenic variations in the *HDG* gene which encodes the homogentisate 1,2-dioxygenase, an enzyme that acts downstream to the conversion of phenylalanine into tyrosine. In AKU, there is an increased serum and urinary levels of homogentisic acid. The accumulation of homogentisic acid, which has an “ochre-like” color in microscopy, is responsible for the formation of ochronotic bluish-black pigmented polymers which have a strong affinity for collagen that creates damage in all connective tissues. The transmission of AKU is autosomal recessive. Its prevalence is 1/1,000,000. Diagnosis is confirmed by high levels of homogentisic acid in the urine [18].

In AKU, there is a latency period before the onset of symptoms. This is known as the pre-ochronotic phase, with slow subclinical evolution followed by a rapidly progressive ochronotic phase [19]. On average symptoms develop at the age of 30–40 years. The initial symptomatology is dominated by chronic mechanical or inflammatory low back pain and spinal stiffness with radiological diffuse calcifications of the intervertebral discs [20, 21]. Secondly, within a period of about a decade, peripheral arthralgia appears. Large joints such as knees, hips or shoulders are preferentially affected. Episodes of joint effusion are possible. Other clinical aspects suggestive of AKU are dark-colored urine when alkalized or oxidized in air, bluish discoloration of the nose, ears and sclera. Ear examination can lead to the diagnosis when it discloses a contrast between a black tympanic membrane and discolored pale cerumen. Other possible affections are obstruction of the urinary tract, renal failure, aortic and mitral valve disease, conductive hearing loss and osteopenia [22, 23].

Synovial fluid is mechanical or inflammatory without microcrystals. Presence of homogentisic acid crystals have been reported. X-rays show joint destruction with narrowing joint space, osteophytosis, osteosclerosis,subchondral geodes and diffuse calcifications of the intervertebral discs [24, 25]. During surgery or arthroscopy, cartilage and synovial membranes are black in color [26].

AKU is treated with nitisinone, a competitive inhibitor of 4-hydroxyphenyl dioxygenase, enzyme of the tyrosine catabolism pathway, thereby preventing the accumulation of toxic intermediate products. An observational, long-term follow up study (SONIA-2) of patients treated with nitisinone at a dose of 10 mg daily has shown that nitisinone decreased ochronosis and improved clinical symptoms, indicating a slower disease progression [4]. Dietary protein restriction, low in phenylalanine and tyrosine, is effective in patients who are below 12 years old but the effectiveness of such diet has not been shown in older patients [27]. Joint replacement surgery has been described as successful in patients with alkaptonuria, although they may carry an higher risk of complication as compared to other patients [28].

#### Mucopolysaccharidoses (moderate forms)

Mucopolysaccharidoses (MPS) are lysosomal storage disorders in which the enzyme deficiency is responsible for intra-lysosomal accumulation of glycosaminoglycans (GAG) that causes systemic tissue dysfunction. This group of disorders presents a great heterogeneity in clinical presentation. Indeed, there are 12 types of MPS in which we will discuss 5 types that may initially occur through isolated joint symptoms in their moderate form. These are MPS 1 or *Scheie syndrome*, MPS 2 or *Hunter syndrome*, MPS 4a or *Morquio A syndrome*, MPS 6 or *Maroteaux-Lamy syndrome* and MPS 7 or *Sly syndrome*. The inheritance of MPS is autosomal recessive, except for MPS 2 which is X-linked. The combined prevalence of all MPS is 1/22,000. The diagnosis of MPS is confirmed by a high concentration or an abnormal urinary GAG profile and by specific enzyme assay. This assay should be carried out despite an analysis of normal urinary GAGs if the patient has symptoms attributable to MPS [29].

The main initial manifestation of MPS moderate forms is progressive joint stiffness and contracture, often starting within the first decade. The affected joints are mainly the hands and shoulders, without inflammatory signs. Hip dysplasia, kyphoscoliosis, multiple dysostosis, short stature, spinal deformities and cervical cord compression are also described. In MPS 4, joint hypermobility is observed and its occurrence at a young age should systematically suggest such diagnosis., especially since the other symptoms are often poor. The presence of muscle-tendon contractures manifested by “claw hand” is characteristic of MPS 1 [30–32]. Non-articular clinical manifestations are recurrent inguinal and umbilical hernia, corneal clouding, mental retardation, facial dysmorphia, hepatosplenomegaly, valvular diseases, hearing loss, sleep apnea syndrome, infections and repeated respiratory tract infections [29].

Treatment for these moderate forms consists in enzyme replacement therapy available for MPS 1, 2, 4a, 6 and 7. Orthopedic surgery may improve quality of life and function [33]. Early diagnosis of these moderate forms by rheumatologist is essential since specific treatment is now available for some subtypes and might, if given early, slow down the development of tissue damage, which is unfortunately irreversible [30].

#### Type 3 mucolipidosis : pseudo-hurler polydystrophy

Type 3 Mucolipidosis is a lysosomal disease due to the N-acetylglucosaminyl-1-phosphotransferase deficiency, an enzyme responsible for targeting lysosomal enzymes to lysosome through the mannose-6-phosphate (M6P) signal. It can be caused by biallelic pathogenic variants in the *GNPTAB* gene (Mucolipidosis type 3-alpha/beta) or in the *GNPTG* (Mucolipidosis type 3-gamma). The inheritance is autosomal recessive. Diagnosis is confirmed through the detection of the increased activity of acid hydrolases in serum and their corresponding decrease in fibroblasts. Treatment is only symptomatic [34].

For Mucolipidosis type 3-gamma, the clinical presentation is close to *Scheie syndrome* (MPS 1), but with no/mild corneal clouding. There is an initial predominant articular symptomatology such as progressive joint stiffness which often begins within the first decade and primarily affects hands and shoulders without any inflammatory signs. The disease can also affect hips and the temporomandibular joint [35, 36]. Other musculoskeletal manifestations are carpal tunnel syndrome, dysostosis multiplex, short stature, spinal deformities and cervical cord compression (with odontoid dysplasia and atlanto-axial dislocation). Other clinical manifestations are hepatosplenomegaly, valvular diseases, deafness, moderate psychomotor retardation and mild facial dysmorphia [5, 34].

#### Type 1 gaucher disease

Gaucher disease (GD) is a lysosomal storage disease of the sphingolipidoses group, due to beta-glucocerebrosidase deficiency associated with *GBA* gene mutations. This deficit is responsible for an intra-lysosomal accumulation of a glycosphingolipid, glucocerebroside, in tissue macrophages. GD type 1 is the non-neurological form, diagnosed in adolescence or adulthood. In the GD French registry, the mean age at diagnosis was 22 years with extremes ranging from 0 to 84 years [37]. The transmission of the disease is autosomal recessive. Its prevalence is 1/50,000 to 1/40,000. Diagnosis is based on the detection of a deficient enzyme glucocerebrosidase activity in leukocytes followed by genetic analysis.

Patients present with pain that mimic joint manifestation, but which correspond to epiphyseal bone infarction known as “bone crisis”, affecting mainly the femoral heads, lumbar vertebrae, the proximal portions of the tibia and humerus. Bone infarction can mimic osteomyelitis and infection should be ruled out. Such infarctions may further lead tosecondary osteoarthritis or mechanical spinal pain associated with post fracture hyperkyphosis. Other musculoskeletal manifestations are the occurrence of pathological fractures on osteoporosis or small in size lytic lesions [38].

The abnormalities that can be identified in X-rays (secondary to epiphyseal bone infarction) look like an Erlenmeyer flask with metaphyseal diaphyseal widening of long bones predominant in distal femoral or proximal tibial and cortical thinning. X-ray may also show localized areas of condensation also known as “bone in the bone” (calcified sequelae of ischemic areas) and sometimes localized pseudo-tumoral lytic foci wrongly suggestive of suspected bone lesions [39]. Systemic manifestations of the disease are asthenia, hepatosplenomegaly, anemia and thrombocytopenia, dysglobulinemia or multiple myeloma and, rarely, interstitial lung disease or portal hypertension.

Therapy of GD is based upon enzyme replacement therapy [40–42] or substrate reduction therapy along with symptomatic measures (including hip arthroplasty if necessary) [43]. Regular MRI follow-up of the femoral heads, osteodensitometry and X-rays of the symptomatic areas are primordial. Because secondary osteoarthritis and osteoporotic fractures occurs in at least 20% of the patients, it is important for rheumatologists to detect and treat GD as earlier as possible.

### Arthritis

Arthritis, destructive or not, microcrystalline or otherwise, may also reveal an IMD in adulthood. Thus, any arthritis, including any crystal-induced arthritis occurring at a young age, should suggest the diagnosis of IMD. Etiological orientation requires an enhanced history taking, including family history, age of onset of symptoms and their evolution and attendant symptoms (Table 2). The type of arthritis should also be able to guide the diagnosis. Crystal-induced arthritis with uric acid crystals may be secondary to a so-called “enzymopathic” gout, that with calcium pyrophosphate crystals secondary to Gitelman syndrome or hypophosphatasia, and that with oxalate crystals to primary hyperoxaluria. The clinician should therefore always look for associated renal impairment. Non-destructive recurrent acute arthritis may suggest a mevalonate kinase deficiency.

**Table 2:** IMDs revealed by arthritis in adulthood: the essential

	Gene	Inheritance	Patients profile rheumatologists should be aware of	Joint manifestations	Treatment
**Enzymopathic gout**	*HPRT* *PRPS*	X-linked recessive	Severe gout in young patients, tophi and urate nephropathy ↓ HGPRT activity ↑ PRPP synthetase activity	Acute mono or polyarthritis of the large joints Deforming asymmetric chronic mechanical polyarthralgia	Urate-lowering drug (allopurinol) Colchicine
**Gitelman**	*SLC12A3*	Autosomal recessive	Muscle weakness, paresthesia, abdominal pain, chondrocalcinosis in young patients. Hypokalemia, hypomagnesemia, hypocalciuria	Chondrocalcinosis	Symptomatic
**Hypophosphatasia**	*ALPL*	Autosomal recessive or dominant	Chondrocalcinosis in young patients, Bone demineralization ↓ serum ALP activity ↑ urinary phosphoethanolamine	Chondrocalcinosis	Symptomatic Teriparatide Monoclonal anti sclerostin antibody,asfotase alpha
**Primary hyperoxaluria**	*AGTX*	Autosomal recessive	Onset before 25 years : microcrystalline arthropathy, urinary lithiasis, vascular, cardiac and eye affection Hyperoxaluria, hyperglycolaturia ↓ AGT activity on PBH	Microcrystalline arthropathy: acute or chronic mono- or polyarthritis predominantly distal	Pyridoxine phosphate, lumisaran
**Wilson disease**	*ATP7B*	Autosomal recessive	Onset before the age of 20 years : Arthritis and chondrocalcinosis, hepatic and neurological form, tubulopathy, osteoporosis, cardiopathy, hemolysis ↓ serum ceruloplasmin ↑ cupruresis	Destructive arthritis of the large joints, Chondrocalcinosis	Copper chelating agents or zinc
**MVK deficiency**	*MVK*	Autosomal recessive	Relapsing fever for 3–7 days, arthritis. ↑ mevalonaturia during an attack	Nonerosive symmetrical arthritis or polyarthritis of the large joints	Symptomatic
**Familial hypercholesterolemia**	*LDLR* *APOB* *PCSK9*	Autosomal dominant	Around the age of 20 years : Tendon xanthomas, coronary artery disease, arthritis ↑ LDL cholesterol	Achilles tendonitis Acute mono or oligoarthritis of the knee / ankle, nondestructive febrile subacute polyarthritis	Statin - ezetimibe Anti-PCSK9
**Sitosterolemia**	*ABCG5* *ABCG8*	Autosomal recessive	Young adult with tendon xanthomas, arterial disease, hepatic or hematological manifestations ↑ Sitosterol	Arthralgias or arthritis Achille tendonitis	Ezetimibe

#### “Enzymopathic” gout

Gout can be secondary to a purine metabolism disorder such as hypoxanthine-guanine phosphoribosyltransferase (HPRT) deficiency, which can be partial or complete in the *Lesch-Nyhan syndrome*. The transmission of these diseases is X-linked. The biological profile includes hyperuricemia (usually greater than patients with primary gout) with hyperuricosuria. Diagnosis is made by measuring the activity of the enzyme on erythrocytes and fibroblasts in culture [44].

HPRT deficiency combines neurological and behavioral disorders with uric acid overproduction symptoms as gout or recurrent nephrolithiasis that may lead to renal failure. Clinical symptoms usually appear in the first years of life but some moderate forms exist without neurological manifestations with the first symptoms onset in adolescence or adulthood up to 30 years. Joint manifestations are the same as those of primary gout but onset is earlier, causing severe chronic urate arthropathy with significant joint deformity. Similarly, patients present with early onset urate nephropathy with recurrent urinary lithiasis and early evolution to renal impairment [45, 46].

Treatment is similar to primary gout, based on allopurinol and symptomatic measures. Since gout in the young adult can sometimes be the only symptom of the disease, rheumatologists must be aware of this condition, especially because these young patients can unknowingly pass the disease to their offspring [44].

#### Gitelman syndrome

Gitelman syndrome, also known as familial hypokalemia-hypomagnesemia, is well known by nephrologists and is due to a mutation of the *NCCT*/*SLC12A3* gene encoding the Na-Cl cotransporter sensitive to thiazide diuretics located at the distal convoluted tubule. The transmission of the disease is autosomal recessive. Its prevalence is approximately 1/40,000. Biological abnormalities lead to this diagnosis: hypokalemia, hypomagnesemia, metabolic alkalosis, hypocalciuria, urinary sodium loss despite an elevation of renine-angiotensine serum levels. The diagnosis is confirmed by genetic analysis. Acquired Gitelman syndrome can also occur in patients with primary Sjögren syndrome [47, 48].

In most cases, symptoms begin after the age of six years and the disease is usually diagnosed during adolescence or adulthood. Symptoms are poor, marked by transient episodes of muscle weakness and cramps, paresthesia, and sometimes abdominal pain and vomiting. Arterial blood pressure is low. The joint manifestation is represented by chondrocalcinosis and is directly linked to hypomagnesemia [47]. Joint manifestations may start before the age of 50 years, with destructive or non-destructive polyarthritis, which develops by recurrent attacks known as “pseudo-gout” [49]. After the age of 50 years, another clinical form is described with more aggressive oligoarticular presentation leading to destructive chronic microcrystalline arthropathy [50].

Treatment is symptomatic with magnesium and potassium supplementation. The treatment for joint crises is the same as that of idiopathic pyrophosphate arthropathy. Rheumatologists should consider this metabolic disorder when chondrocalcinosis occurs in younger patients.

#### Hypophosphatasia

Hypophosphatasia is caused by a deficiency in the activity of serum alkaline phosphatase due to *ALPL* gene mutations. The mode of transmission may be autosomal recessive or dominant. The diagnosis is made by a sharp decline in serum alkaline phosphatase activity after excluding other diseases that may reduce serum alkaline phosphatase. Biological assessment may show hypercalcemia and hypercalciuria. *ALPL* gene testing confirm the diagnosis [51].

There is a wide clinical variability. Several clinical forms are currently recognized: pediatric forms, adult form and odontohypophosphatasia. The adult form is characterized by pyrophosphate arthropathy associated with bone demineralization manifesting as multiple fractures. Non-specific muscle and bone pain is often reported. Calcified periarthritis due to apatite crystal deposition may occur. Calcium deposits in skin, nose and fingers are described. Delayed bone healing and dental disorders with cavities, tooth fractures or early loss of teeth are often associated. Other signs include myopathy, nephrocalcinosis and ophthalmic calcification [52, 53].

X-ray examination is non-specific and may show bone demineralization, osteomalacia-like changes, bowed long bones, epiphyseal lucencies, osteophytises specifically in the fibula and the ulna, fractures and pseudo-fractures and chondrocalcinosis [51].

Enzyme replacement therapy by asfotase alpha is available but dedicated to the most severe pediatric forms. Teriparatide has been shown to improve pain, mobility and fracture repair in adult patients. Monoclonal antisclerostin antibodies has been shown to increase bone mineral density in patients with hypophosphatasia [51, 54].

Fractures linked to hypophosphatasia can be confused with osteoporotic fractures, thus rheumatologists should adjust treatment in consequence. Indeed, bisphosphonate and denosumab may worsen the disease and vitamin D supplementation may induce and aggravate hypercalcemia [51].

#### Primary hyperoxaluria

There are 3 types of primary hyperoxaluria or oxalosis. Type 1 is the most common (80%) and is related to deficiency in a hepatic peroxisomal enzyme, alanine glyoxylate aminotransferase (AGT), in relation with *AGTX* gene mutations. The transmission of the disease is autosomal recessive. The prevalence is 1-2.9/1 000 000. There are secondary causes of hyperoxaluria in patients with fat malabsorption following bariatric surgery procedures, or chronic pancreatitis. Diagnosis is suggested by the presence of hyperoxaluria or in case of kidney failure with nephrocalcinosis. It is confirmed by genetic analysis with possible prenatal diagnosis, or by measuring AGT activity on liver biopsy [55].

Symptoms usually appear between 1 and 25 years but the diagnosis is often made later. The first symptoms are the occurrence of recurrent urinary lithiases, complicated by progressive renal impairment due to nephrocalcinosis. Oxalate accumulates within the tissues and causes multisystem disease with hypertension, arterial disease of the limbs, anemia, heart rhythm disorders, ocular signs [56].

From a joint perspective, primary hyperoxaluria is responsible for microcrystalline arthropathy which is expressed as arthralgias, acute or chronic predominantly distal symmetric polyarthritis (proximal interphalangeal and metacarpophalangeal joints, knees, elbows and ankles), tenosynovitis, bursitis and/or calcium skin deposits. X rays reveal calcification, pyrophosphate arthropathy, fractures and pseudofractures, sclerosis, cystic bone changes, dense metaphyseal bands and subperiosteal resorption adjacent to oxalate deposits. Synovial fluid shows some polymorphic intracellular oxalate crystals [57].

Treatment of primary hyperoxaluria relies on the prevention of complications with hyper-hydration, urine alkalinization, dietetic measures and pyridoxine phosphate intake (vitamin B6, the AGT coenzyme). The only curative treatment is liver transplantation with or without kidney transplantation. New RNA therapy lumisaran, has been approved in Europe and USA [58, 59]. Diagnosis is made early in only 30% patients although undelayed diagnosis is the key because the effectiveness of the treatment depends on early onset [55, 57].

#### Wilson’s disease

Wilson’s disease (WD) is a copper metabolism disorder due to mutations of the *ATP7B* gene encoding for a hepatocyte copper carrier that induces an inability of the liver cell to evacuate copper as well as a significant decrease in the synthesis of circulating ceruloplasmin. This results in liver tissue copper accumulation and then extrahepatic accumulation by releasing free copper into the general circulation. The disease is transmitted in an autosomal recessive mode. Its prevalence is estimated at 1/30,000 [60].

Diagnosis is difficult and is based on a range of clinical and biological biomarkers. This is a combination of a low or collapsed serum ceruloplasmin and high urinary copper concentrations. Rarely, a liver biopsy to measure copper levels can be useful [61]. Genetic analysis is performed for diagnosis confirmation or family screening but the negativity of the test does not rule out the diagnosis even with recent progress on the subject [62].

The onset of clinical manifestations is from 5 to 35 years and clinical presentation varies widely from one individual to another. The revealing symptoms are often hepatic or neurological. The hepatic form is revealed by acute hepatitis, but may also present as chronic hepatitis or cirrhosis. The neurological form gradually associates tremor, dysarthria, dystonia, swallowing disorders and psychiatric symptoms in varying degrees. The late onset form is characterized by a significant liver injury but relatively mild neurological symptoms [60, 63].

In case of neurological impairment, brain MRI shows pronounced T1 hypointense lesions and T2 hyperintense lesions affecting the basal ganglia. Mesencephalic damage gives a “Face of the Giant Panda” appearance. Other possible manifestations of WD are hematological hemolysis, renal tubulopathies, osteomalacia and osteoporosis and cardiomyopathies with arrhythmias. A Kayser-Fleischer ring can be observed by slit lamp biomicroscopy [60].

WD can also manifest through joint symptoms that usually appear after the age of 20 years such as destructive symmetric arthritis of the large joints, affecting spine, wrists, elbows, shoulders, hips, knees and metacarpophalangeal joints. X-rays show joint damage with joint space narrowing, osteophytes, subchondral sclerosis, subchondral geodes, periarticular osteopenia. Pyrophosphate arthropathy can occur in some cases [64, 65].

Treatment is based on the use of either copper chelators such as D-penicillamine or triethylenetetramine, or zinc which reduces copper absorption [66]. An unexplained polyarthritis in a patient with liver and neurological symptoms should push the clinicians to screen for Wilson’s disease.

#### Mevalonate kinase deficiency

Mevalonate kinase deficiency (MKD), formerly known as “Hyper-IgD syndrome”, is related to a deficiency in an enzyme involved in cholesterol biosynthesis that results in the accumulation of mevalonic acid. Transmission is autosomal recessive and the mutation affects the *MVD* gene. Diagnosis is mainly clinical but biological arguments can be found by detection of urinary mevalonic acid during febrile attacks or dosing lymphocytic mevalonate kinase activity. An increased polyclonal IgD and IgA serum levels is present in most patients during the crisis along with an important elevation of C-reactive protein and/or neutrophilic leukocytosis. The search for a mutation in the gene encoding *MVK* is then carried out to confirm diagnosis [67].

MKD is a systemic autoinflammatory disease (SAID) defined as abnormal activation of innate immunity in the absence of autoimmunity. Because of their genetic nature, these diseases often have an early onset, but some patients can develop symptoms during adulthood. They are rare diseases and clinical spectrum is wide. Some SAID are characterized by recurrent flares of systemic inflammation associating fever, rash, serositis and lymphadenopathy separated by symptom-free intervals. Others present a more chronic course [68].

For the rheumatologist, SAIDs can mimic autoimmune disease. Early onset, family history, absence of auto-immunity, acute inflammatory flares and no effect of conventional disease-modifying anti-rheumatic drugs (cDMARDs) should raise suspicion.

Concerning MKD, the moderate adult form is characterized by a clinical picture dominated by recurrent febrile crises that begin in the first years of life, with a duration of 3 to 7 days every 2 to 8 weeks, with a frequency that decreases with age. Patients remain asymptomatic outside crisis. During acute crisis, they may suffer from, in order of frequency, asthenia, lymphadenopathy (that can be painful), abdominal pain, arthralgia, maculopapular rash, headache, pharyngitis, uveitis mood disorders and sometimes from severe neurological symptoms. Complications are AA amyloidosis, joint stiffness and abdominal adhesions [67, 69].

Acute MKD crises may be accompanied by inflammatory polyarthralgia with or without arthritis, non-erosive, symmetric, affecting large joints, especially knees and hips and sometimes metacarpophalangeal joint [69].

Prognosis of MKD is usually good, carrying a normal life expectancy, although the quality of life is significantly affected. The treatment strategy consists in on-demand therapy during flares by corticosteroids or anti-IL1 agents (canakinumab). A continuous therapy is used in most patients depending on the evolution. Anti-TNF alpha may also be effective [70].

#### Heterozygous familial hypercholesterolemia

Familial hypercholesterolemia (FH) is a lipid metabolism disorder characterized by a sustained elevation of circulating low density lipoprotein (LDL), by default of endocytosis, linked with mutations in the encoding genes for LDLR, APOB and PCSK9 proteins. There are two main clinical forms: a less noisy heterozygous form and a more severe homozygous form. Transmission is autosomal dominant. Prevalence is estimated at 1/500 in the heterozygous form. The heterozygous form is characterized by an LDL cholesterol of around 3 g/L (7–8 mmol/L). The Dutch lipid score is commonly used for the probability estimation of FH [71].

In the heterozygous form, tendon xanthomas, subcutaneous xanthomas at the elbow, corneal arch and xanthelasma (eyelid xanthoma) appear within the second and third decade, and finally arterial disease often appears after the age of 30 years. In the homozygous form, the same manifestations are seen earlier, with cutaneous xanthomas and manifest arterial disease before the age of 10 years [72].

Joint manifestations occur in 40% these patients affected by FH, and begin around the age of 18–20 years. The main manifestation is represented by recurrent episodes of subacute pain in the Achilles tendons, responsible for a limp, with or without signs of localized inflammation, spontaneously evolving favorably within two-three days [73]. Some old reviews mention episodes of arthritis spontaneously evolving favorably within ten days without joint destruction [74].

Treatment for heterozygous FH consists of a lipid-lowering diet and treatment aimed at reducing LDL cholesterol by 50% with the prescription of statin +/- ezetimibe. Two PCSK9 inhibitors, evolucumab and alirocumab, may be prescribed in patients who do not reach the LDL-cholesterol target at the maximum tolerated dose of statin [72, 75]. The aim of the treatment is mainly to prevent ischemic heart disease and early clinical identification of tendon xanthomas is essential in that way [76]. Usually, tendon xanthomas resolves after the start of the lipid-lowering treatment [73].

#### Sitosterolemia

Sitosterolemia, also known as phytosterolemia, is an autosomal recessive lipid metabolism disorder resulting in increased serum concentrations of plant sterols. Sitosterolemia is linked with mutations of *ABCG5* and *ABCG8* gene (that play an important role in excreting sterols from the liver and intestine). Prevalence is roughly estimated at 1 in 200 000 (but is difficult to assess because patients are often misdiagnosed as FH). Diagnosis is done measuring serum sitosterol and is confirmed if FH is eliminated by genetic analysis [77].

Clinical manifestations are globally similar to FH, but with a wide variability, and usually appear in young adults. Tendon xanthomas and subcutaneous xanthomas are often described as the first manifestations of the disease. Cytopenia with or without splenomegaly and hepatic fibrosis can happen. In the end, without treatment, arterial disease usually appears [78]. Arthralgias occur with recurrent episodes and may be misdiagnosed as an inflammatory rheumatic disease. Few details about joint manifestations are available given to the rarity of the disease [79, 80].

Tratment for sitosterolemia consists in a plant sterol lowering diet, with lipid lowering treatment. Statins are often ineffective, and ezetimibe is the main molecule to use. An optimal treatment results in prevention of arterial disease and can help to reduce joint manifestations [77].

## Conclusions

Joint manifestations in adults, such as arthralgias, epiphyseal pain, joint stiffness, periarthritis or arthritis may help discover late-onset IMD. The clinical presentation of these essentially pediatric diseases, when they occur in adulthood, can then take unexpected forms. Lack of knowledge of adult physicians leads to an underestimation of their frequency and management that is often delayed, with potentially severe rheumatological or systemic sequelae. In addition, considerable progress has been made in the treatment of IMD in recent years, with the development of more targeted therapies, hence the importance of identifying them the earlier as possible. When such diagnosis is suspected, it is advisable to refer patients to specialized centers for the management of IMD.

### Electronic supplementary material

Below is the link to the electronic supplementary material.

Supplementary Material 1

## Data Availability

Not applicable.

## List of Abreviations

IMD: Inborn metabolic diseases
HH: Hereditary Hemochromatosis
AKU: Alkaptonuria
MPS: Mucopolysaccharidoses
GAG: Glycosaminoglycans
GD: Gaucher disease
HPRT: Hypoxanthine-guanine phosphoribosyltransferase
AGT: Alanine glyoxylate aminotransferase
WD: Wilson’s disease
MKD: Mevalonate kinase deficiency
SAID: Autoinflammatory disease
FH: Familial hypercholesterolemia
LDL: Low density lipoprotein
