# Supplementary material for: Joint manifestations revealing inborn metabolic diseases in adults: a narrative review
Source: Orphanet J Rare Dis. 2023 Aug 10;18:239. doi: 10.1186/s13023-023-02810-6 (PMC10416490; doi:10.1186/s13023-023-02810-6)
Supplement: Supplementary file 1 — Supplementary Material 1 [file 13023_2023_2810_MOESM1_ESM.docx]

To: Francesc Palau, *Hospital Sant Joan de Déu Barcelona and CIBERER, Spain*

*Editor in chief - Orphanet Journal of rare diseases*

Dear Editor,

Please find a manuscript entitled “Joint Manifestations Revealing Inborn Metabolic Diseases in Adults: a narrative review” which we submit for publication in *Orphanet Journal of rare diseases*. The present paper is an extensive review of the literature which summarizes the inherited metabolic that can be diagnosed in adult patients with joint pain or arthritis. These diseases are considered as rare but they are underdiagnosed, specifically in adults. Our paper may then help clinicians to suspect and diagnose inherited metabolic disease in their clinical practice, and treat the affected patients as more treatments are actually getting available.

For all these reasons, we hope that our paper could be of interest for the readers of *Arthritis Care and Research.* Please note that this review is an original work and was not submitted elsewhere. All authors have approved the manuscript for submission.

Best Regards,

Amaury LORET

François MAILLOT
